# The Dark Side of Pyroptosis of Diffuse Large B-Cell Lymphoma in B-Cell Non-Hodgkin Lymphoma: Mediating the Specific Inflammatory Microenvironment

**DOI:** 10.3389/fcell.2021.779123

**Published:** 2021-11-05

**Authors:** Wei Wang, Shi-wen Xu, Ya Teng, Min Zhu, Qun-yi Guo, Yuan-wen Wang, Xin-Li Mao, Shao-wei Li, Wen-da Luo

**Affiliations:** ^1^ Taizhou Hospital of Zhejiang Province Affiliated to Wenzhou Medical University, Linhai, China; ^2^ Department of Hematology, Taizhou Hospital of Zhejiang Province Affiliated to Wenzhou Medical University, Linhai, China; ^3^ Key Laboratory of Minimally Invasive Techniques and Rapid Rehabilitation of Digestive System Tumor of Zhejiang Province, Taizhou Hospital of Zhejiang Province Affiliated to Wenzhou Medical University, Linhai, China; ^4^ Department of Gastroenterology, Taizhou Hospital of Zhejiang Province Affiliated to Wenzhou Medical University, Linhai, China; ^5^ Institute of Digestive Disease, Taizhou Hospital of Zhejiang Province Affiliated to Wenzhou Medical University, Linhai, China

**Keywords:** pyroptosis, diffuse large B-cell lymphoma, tumor immune microenvironment, inflammation, prognosis

## Abstract

**Background:** Diffuse large B-cell lymphoma (DLBCL) is a common aggressive B-cell non-Hodgkin lymphoma (B-NHL). While combined chemotherapy has improved the outcomes of DLBCL, it remains a highly detrimental disease. Pyroptosis, an inflammatory programmed cell death, is considered to have both tumor-promoting and tumor-suppressing effects. The role of pyroptosis in DLBCL has been gradually appreciated, but its value needs further investigation.

**Methods:** We analyzed mutations and copy number variation (CNV) alterations of pyroptosis-related genes (PRGs) from The Cancer Genome Atlas (TCGA) cohort and evaluated the differences in expression in normal B cells and DLBCL patients in two Gene Expression Omnibus (GEO) datasets (GSE12195 and GSE56315). Based on the expression of 52 PRGs, we divided 421 DLBCL patients from the GSE31312 dataset into distinct clusters using consensus clustering. The Kaplan-Meier method was used to prognosis among the three clusters, and GSVA was used to explore differences in the biological functions. ESTIMATE and single-sample gene-set enrichment analysis (ssGSEA) were used to analyze the tumor immune microenvironment (TME) in different clusters. A risk score signature was developed using a univariate survival analysis and multivariate regression analysis, and the reliability and validity of the signature were verified. By combining the signature with clinical factors, a nomogram was established to predict the prognosis of DLBCL patients. The alluvial diagram and correlation matrix were used to explore the relationship between pyroptosis risk score, clinical features and TME.

**Results:** A large proportion of PRGs are dysregulated in DLBCL and associated with the prognosis. We found three distinct pyroptosis-related clusters (cluster A, B, and C) that differed significantly with regard to the prognosis, biological process, clinical characteristics, chemotherapeutic drug sensitivity, and TME. Furthermore, we developed a risk score signature that effectively differentiates high and low-risk patients. The nomogram combining this signature with several clinical indicators showed an excellent ability to predict the prognosis of DCBCL patients.

**Conclusions:** This work demonstrates that pyroptosis plays an important role in the diversity and complexity of the TME in DLBCL. The risk signature of pyroptosis is a promising predictive tool. A correct and comprehensive assessment of the mode of action of pyroptosis in individuals will help guide more effective treatment.

## Introduction

B-cell non-Hodgkin lymphoma (B-NHL), which accounts for approximately 85–90% of non-Hodgkin lymphoma, is a group of diseases with significant heterogeneity. Diffuse large B-cell lymphoma (DLBCL), representing about 30% of non-Hodgkin lymphomas cases, is an important subset of aggressive B-NHL. At present, as a first-line treatment, rituximab plus cyclophosphamide, doxorubicin, vincristine, prednisone (RCHOP) has improved the prognosis of DLBCL patients, and about 65% of such patients achieve relief at the initial treatment. However, the final the prognosis of the disease is still not optimistic (Coiffier et al., 2010; Shankland et al., 2012; Dunleavy and Gross, 2018; Sehn and Salles, 2021).

Pyroptosis is a kind of programmed cell death in the form of inflammation, and Gasdermin D (GSDMD) is considered the main executor (Shi et al., 2017). Early studies have clarified the important role of pyroptosis and related proteins in the process of fighting infection. Moderate pyroptosis contributes to the stability of the intracellular environment, effectively prevents excessive cell proliferation, and protects the host (Aglietti and Dueber, 2017). However, more and more studies have proven that pyroptosis plays an important role in tumors, and this role is bidirectional. Pyroptosis can regulate malignant phenotypes, such as cell morphology, proliferation, infiltration, migration, and chemotherapy tolerance, through a variety of cell signal pathways, to affect the progress of tumors and has been shown to be related to the patient prognosis (Xia et al., 2019). The expression of NLRP1 in colorectal cancer was decreased compared with normal tissues, and a higher tumor incidence was observed in NLRP1^−/−^ mice (Williams et al., 2015). The expression of NLRP3 decreased or was even lost in hepatocellular carcinoma (HCC), which is related to poorer pathological differentiation and advanced stage, suggesting that the gene may be a tumor suppressor gene in HCC (Wei et al., 2014). However, in pancreatic ductal adenocarcinoma, interleukin (IL)-10-dependent NLRP3 signaling was shown to be involved in inducing immunosuppressive microenvironment formation by promoting tumor-associated macrophage expansion (Daley et al., 2017).

A large number of inflammatory mediators are released during pyroptosis, and the two most important are IL-1β and IL-18 (Fink and Cookson, 2006). Most studies suggest that IL-1β plays an important role in promoting tumor cell proliferation, invasion, and metastasis. For example, after blocking IL-1β, CD8^+^ lymphocytes in breast cancer tissue were activated, leading to tumor growth restriction (Kaplanov et al., 2019). Through the NF-κB/miR-506/JAG1 signaling pathway and NF-κB/miR-376c/TGFA signaling pathway, IL-1β is involved in promoting the proliferation of osteosarcoma (Hu et al., 2017; Liu et al., 2017). In addition, IL-1β may play a role in the process of epithelial-mesenchymal transition (EMT) (Zhang et al., 2018). IL-18 may play an anti-tumor immune role by increasing the activity of CD8^+^T cells and natural killer cells and promoting IFN-γ anti-tumor activity (Srivastava et al., 2010). Furthermore, IL-18 is also involved in the disease progression and immunosuppression of multiple myeloma (MM), and a high level of IL-18 is associated with a poor prognosis in such patients (Nakamura et al., 2018).

The role of pyroptosis in lymphoma has been receiving increasing attention. The combination of BAFF and BAFF receptor triggered the initiation and activation signal of NLRP3 inflammatory body and induced pyroptosis of the B lymphoma cell line (Lim et al., 2020). NLRP3 inflammasome can induce the anti-dexamethasone effect of lymphoma cells through IL-18, thereby inhibiting apoptosis and promoting tumor proliferation (Zhao et al., 2017). Cancer cells can shape the tumor microenvironment (TME) suitable for their growth by inducing reprogramming of surrounding cells (Hinshaw and Shevde, 2019). The TME and restricted immune surveillance play important roles in the occurrence and development of lymphoma (Autio et al., 2021). The impact of pyroptosis involvement on the TME, however, has not yet been sufficiently studied in DLBCL. Furthermore, current research is largely limited to a single or few pyroptosis-related molecules. Those studies have the same limitation, wherein the antitumor effect or the occurrence and development of the tumor is the result of the interaction of many factors in a highly coordinated way; therefore, individual studies may not reflect the complete role of pyroptosis.

Given the above, we conducted a comprehensive study of pyroptosis in DLBCL by integrating gene expression information and clinical information from multiple datasets. Our analyses explore the impact of pyroptosis on the DLBCL TME and tumor biological behavior and may provide additional evidence for advancing the understanding of DLBCL disease and the direction of treatment.

## Materials and Methods

### Data Download and Processing

The public gene expression databases were downloaded from the Gene Expression Omnibus (GEO, https://www.ncbi.nlm.nih.gov/geo/) database and The Cancer Genome Atlas (TCGA) database (Project name: Lymphoid Neoplasm Diffuse Large B-cell Lymphoma, https://portal.gdc.cancer.gov/projects/TCGA-DLBC). We included GSE31312, GSE10846, GSE12195, GSE56315, and TCGA-DLBC in subsequent analyses. Those four GEO datasets were derived from the same platform (GPL570 Affymetrix Human Genome U133 Plus 2.0). We downloaded raw CEL files and adopted a robust multiarray average (RMA) method to normalize the data. GSE12195 and GSE56315 were used to determine the expression patterns of the pyroptosis-related genes (PRGs) between normal B cells and DLBCL. The results were demonstrated using a box diagram.

The GSE31312 dataset contains abundant clinical information, including the age, gender, survival information, Gene Expression Profiling (GEP), treatment regimen (RCHOP) and response, and the International Prognostic Index (IPI) as the main analysis objects. The GSE10846 dataset, which was also treated using the RCHOP regimen, was used as a validation set. We further processed GSE31312 and GSE10846 as follows: 1) patient sample data with an overall survival time of <30 days were excluded; and 2) for the GSE31312 dataset, samples missing the clinical information mentioned above were excluded.

We obtained information on the somatic mutation and copy number variation (CNV) of DLBCL from the TCGA-DLBC dataset. R package “maftools” was used to analyze the mutation frequency of PRGs and visualize the results using an oncoplot waterfall plot (Mayakonda et al., 2018). We calculated the CNV frequency of PRGs and displayed the results with a lollipop chart. The “RCircos” package of the R software package was used to visualize the location of those genes on chromosomes (Zhang et al., 2013).

### Investigating Different Clusters of DLBCL Based on PRGs

We obtained 52 PRGs from “REACTOME PYROPTOSIS”, “GOBP PYROPTOSIS”, and previous studies (Man and Kanneganti, 2015; Wang and Yin, 2017; Karki and Kanneganti, 2019; Xia et al., 2019; Ye et al., 2021). “REACTOME PYROPTOSIS” and “GOBP PYROPTOSIS” were derived from MSigDB (Molecular Signatures Database v7.4). The R software package “ConsensusClusterPlus” was used to screen the distinct clusters based on the expression of the 52 PRGs (Wilkerson and Hayes, 2010). Resampling was performed 100 times to ensure classification reliability. We then investigated the relationship between clusters and clinical features.

### Identification of Different Biological Processes Between Distinct Clusters

To determine whether or not there were differences in biological processes among different clusters, we downloaded “c2.cp.kegg.v7.4.symbols.gmt” and “h.all.v7.4.symbols.gmt” from MSigDB. The pathways with adjusted *p* values < 0.05 were considered to have significant differences. The above analysis process was performed using the R software package “GSVA” (Hänzelmann et al., 2013). Finally, the significant pathways were presented as a heatmap.

### The Evaluation of the TME

We used the R software package “ESTIMATE” (Yoshihara et al., 2013) to assess the “immunescore” and “stromalscore” in DLBCL. Sngle-sample gene-set enrichment analysis (ssGSEA) was used to quantify estimation of infiltration abundance of different immune cells (Supplementary file). To avoid unnecessary interference, we excluded B cells and related immune cells. Through the ssGSEA algorithm, we obtained the score of each immune cell in each patient and standardized the findings by the Min-Max method. We collected other TME-related signatures through previous studies, including the 1) CD8 T-effector signature (Rosenberg et al., 2016); 2) pan-fibroblast TGFβ response signature (pan-F-TBRS) (Mariathasan et al., 2018); 3) antigen-presenting machinery (APM) signature (Şenbabaoğlu et al., 2016); 4) angiogenesis signature (Şenbabaoğlu et al., 2016); 5) three epithelial mesenchymal transition) (EMT) signatures (EMT1, EMT2, and EMT3) (Damrauer et al., 2014; Hedegaard et al., 2016; Hugo et al., 2016), and 6) two stromal gene signatures (stromal 1 and stromal 2). We then adopted the same process with those signatures (Lenz et al., 2008).

### Establishment of the Prognostic Gene Signature

As the training dataset, the GSE31312 dataset was used to screen for PRGs associated with the prognosis of DLBCL patients. The expression of the PRGs was first determined using a univariate Cox analysis (Cox, 1972). Genes with *p* values < 0.01 were retained. We used a Lasso-penalized Cox regression analysis and Stepwise regression analysis to further screen for PRGs with the best predictive performance and build the risk score signature. The multivariate regression model was then internally validated using the bootstrapping method. After obtaining the regression coefficients for each enrolled gene, we calculated the risk score for each patient based on the expression of each gene using the following formula:

The risk score = (Exp _gene1_ × coefficient _gene1_) + (Exp _gene2_ × coefficient _gene2_) +···+ (Exp _gene7_ × coefficient _gene7_)

Patients were then divided into high and low-risk groups according to the median risk score. Differences in the overall survival (OS) of patients in different risk groups were assessed using Kaplan-Meier curves (Ranstam and Cook, 2017) and AUC of the ROC curve (Kamarudin et al., 2017). The regression coefficients obtained from the training dataset were then applied to the GSE10846 testing dataset with complete clinical information to calculate the patient’s risk score for external validation.

### Construction and Validation of the Predictive Nomogram

To determine whether or not the gene signature has independent prognostic value and to obtain clinically independent prognostic factors associated with the DLBCL prognosis, we performed univariate and multivariate Cox regression analyses on prognostic gene signature and clinicopathological parameters, including the age, gender, GEP, treatment regimen (RCHOP) and response, and IPI, in the training dataset. Factors with *p* < 0.05 in the univariate analysis were selected for a further multivariate Cox regression analysis. The above-incorporated independent parameters were used to construct a prognostic nomogram by stepwise Cox regression to predict the OS in patients with DLBCL at 2, 4, 6, and 8 years. The AUC-ROC curve, Harrell’s concordance index (Harrell et al., 1982) and a calibration plot comparing the predicted and observed OS were used to evaluate the performance of the nomogram.

### Relationship Between the Pyroptosis Risk Score, Clinical Features and TME

An alluvial diagram was developed showing the changes in pyroptosis-related clusters, pyroptosis risk score, IPI score, and treatment response using the R software package “ggalluvial” (Brunson and Read, 2020) and “ggplot2” (Villanueva and Chen, 2019). We performed the R function “cor” to calculate the correlation coefficients between the TME (including infiltrating immune cells and other TME signatures), mRNA expression of IL-1β and IL-18 and pyroptosis risk score, and the correlation matrix was drawn using the “corrplot” package (Wei et al., 2017).

## Results

### Schematic Diagram of the Overall Flow of the Study

**FIGURE 1:**
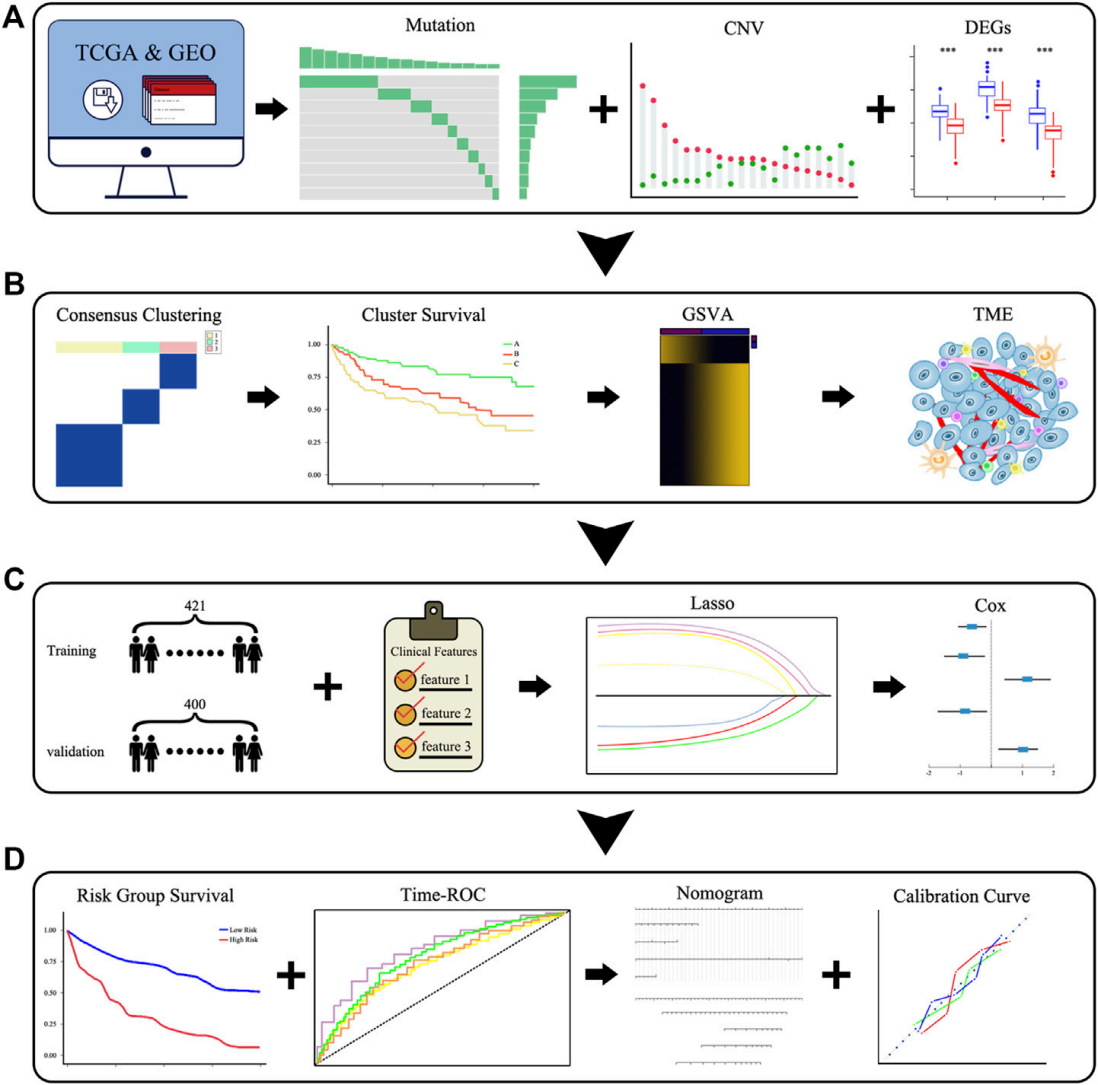
Schematic diagram of the overall flow of the study. **(A)** The landscape of the genetic and expression variation of PRGs. **(B)** Identification of pyroptosis-related clusters and a comparison of the differences in the survival, biological pathways, and TME among clusters. **(C,D)** Identification of pyroptosis-related signatures and construction of a predictive nomogram.

We downloaded the target databases from TCGA and GEO databases and performed preliminary processing of the data, after which we explored the landscape of the genetic and expression variation of PRGs in DLBCL (Figure 1A). We then performed consensus clustering according to the expression of 52 PRGs and compared the differences in the survival, biological processes and TME among different clusters (Figure 1B). Subsequently, we constructed a prognostic signature based on the gene expression and survival information of the 421 patients in the training set. The time-ROC curve was used to evaluate the stability, and external verification was performed to prove the reliability of the signature. We then integrated the clinical features, constructed a predictive nomogram and evaluated the model with calibration curves (Figures 1C,D).

### Landscape of Genetic and Expression Variation of PRGs in DLBCL

**FIGURE 2:**
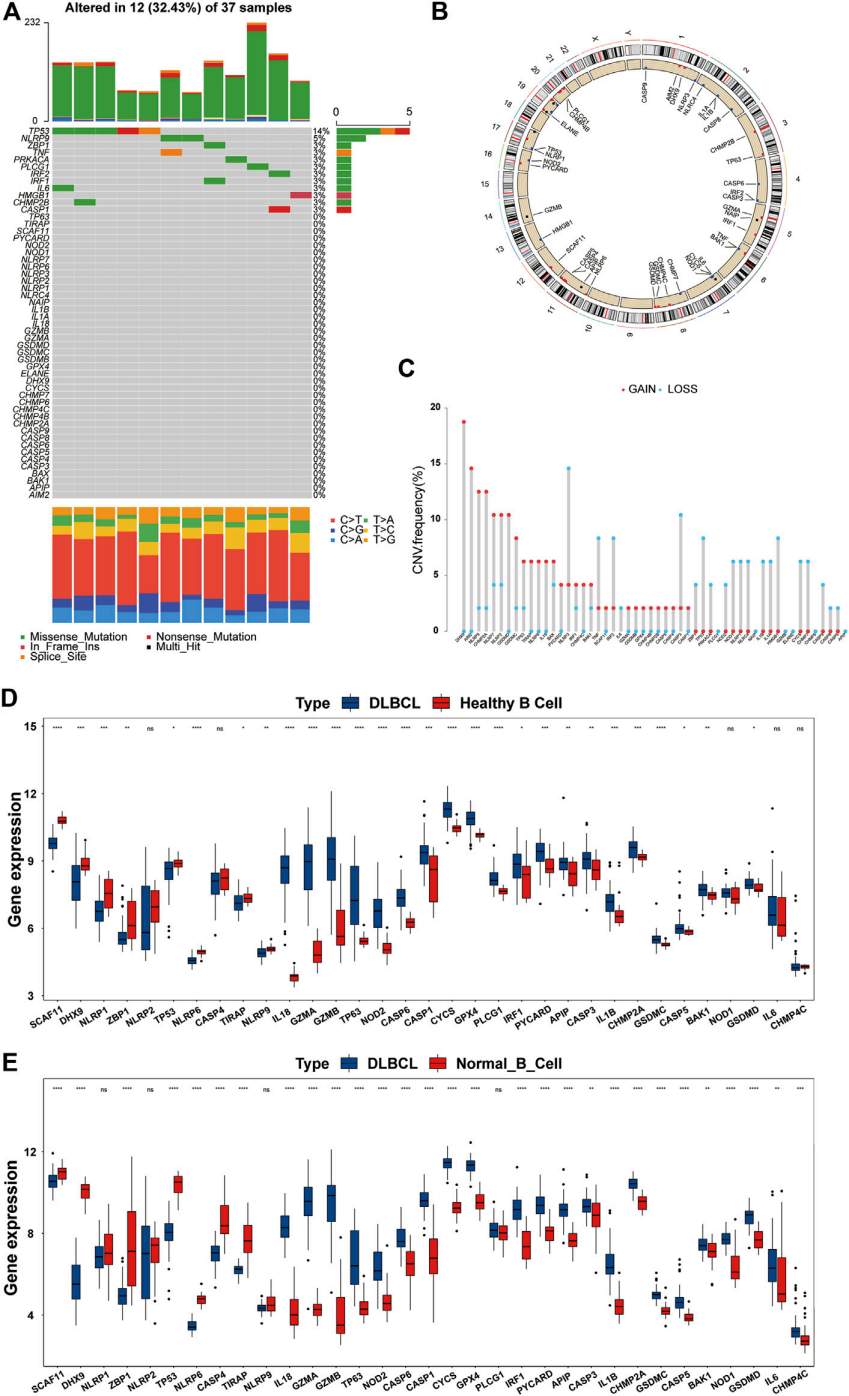
Overview of genetic and expression variation of PRGs in DLBCL. **(A)** The mutation frequency of PRGs in DLBCL patients from the TCGA-DLBC dataset. Each column represents a patient, and each row represents a PRG. Numbers on the **right** represent frequencies. Different colors in the **bottom** annotation represent different mutation types. **(B)** The CNV frequency of PRGs in DLBCL patients from the TCGA-DLBC dataset. The red dot represents amplification, and the blue dot represents deletion. The corresponding height of the column represents the frequency. **(C)** The location of PRGs with CNV information on chromosomes. **(D,E)** Differentially expressed PRGs in normal B cells and DLBCL from the GSE12195 and GSE56315 datasets. DLBCL sample, blue box; normal B cell sample, red box. ns, not statistically significant; *p < 0.05; **p < 0.01; ***p < 0.001; ****p < 0.0001 (all significance designations that appear in this paper are minor criteria).

First, we summarized the incidence of CNV and somatic mutations of the 52 PRGs in DLBCL based on the TCGA-DLBC dataset. Twelve of 37 patient samples had somatic mutations, the most common form being missense mutations. At the gene level, a total of 12 genes were mutated, with TP53 showing the highest frequency (up to 14%), followed by NLRP9; the remaining 10 genes, including ZBP1 and TNF, showed roughly 3% frequency (Figure 2A). To our surprise, GSDMD did not show any mutations in DLBCL samples. By estimating the frequency of CNV, we found that PRGs showed prevalent CNV alterations. However, PLCG1, NAIP, GZMB, ELANE, ELANE, and APIP were neither amplified nor deleted (Figure 2C).

The locations of the CNV alteration of PRGs on chromosomes are shown in Figure 2B. To determine whether or not CNV alteration of PRGs affects the mRNA expression and to ensure the stability of the results, we verified these findings in the GSE12195 and GSE56315 datasets at the same time. We first excluded genes with inconsistent expression trends in the two GEO datasets and then presented the remaining genes in the form of boxplots (Figures 2D,E). Most genes were upregulated in DLBCL, and we also found that among the upregulated PRGs, PYCARD, IRF1, GZMA, GSDMD, GSDMC, GPX4, CHMP2A, CASP5, CASP1, IL-18, and BAK1 showed an amplified CNV status, suggesting a significant relationship between the PRG expression and CNV. After the discovery that the expression of a large proportion of PRGs was imbalanced in normal B cells and DLBCL samples, we analyzed the association of these genes with the prognosis by a univariate Cox regression analysis. As shown in Supplementary Figure S1A, there was a significant correlation between the PRGs and prognosis. These findings suggested that genetic and expression variation of PRGs is common in DLBCL, so pyroptosis may play an important role in the occurrence and development of DLBCL.

### PRG-Related Clusters Identified in DLBCL

**FIGURE 3:**
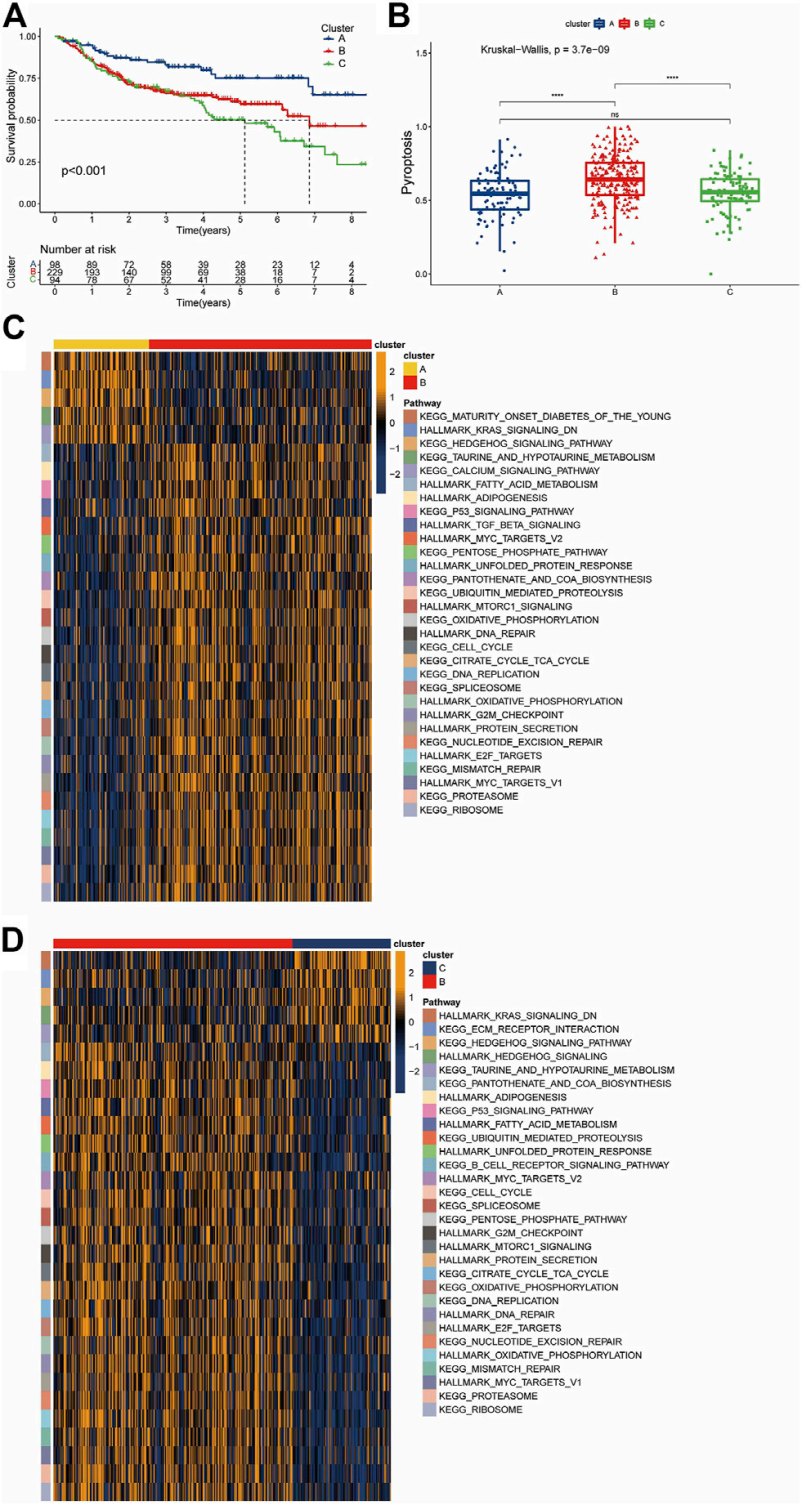
Pyroptosis-associated clusters and differences in biological features. **(A)** Kaplan-Meier survival plots for the three pyroptosis-related clusters. Blue represents cluster A, red cluster B, and green cluster C. **(B)** Pyroptosis signature score differences among the three clusters. Blue represents cluster A, red cluster B, and green cluster C. **(C,D)** The results of a GSVA enrichment analysis are shown as a heatmap. The bars at the top represent different clusters. The square color blocks of different colors on the **right** correspond to the **left**most color block, which represents different paths. In the heatmap, yellow represents pathway activation, and dark blue represents pathway inhibition.

We clustered GSE31312 based on the expression of 52 PRGs quantities by the R package “ConsensusClusterPlus” and ultimately identified 3 completely different clusters, including 98 samples in cluster A, 229 samples in cluster B, and 94 samples in cluster C (Supplementary Figures S1B,C). We subsequently performed a survival analysis of the three clusters and found that they had distinct prognoses, with cluster A having the best prognosis and cluster B having a more favorable long-term prognosis than cluster C (Figure 3A). The ssGSEA method was used to establish a pyroptosis signature score for the three clusters, with results showing that cluster B had the highest score, while there was no significant difference between the scores of A and C (Figure 3B).

Given that the three clusters had different prognoses under the same treatment regimen, we speculated that the clusters were biologically functionally different, and the “GSVA” enrichment analysis validated our hypothesis. Compared with cluster B, cluster A showed enrichment of “KEGG HEDGEHOG SIGNALING”, “HALLMARK KRAS SIGNALING Down” and “KEGG CALCIUM SIGNALING”, among others. For cluster B, a large number of pathways associated with nutrient metabolism were enriched, including “HALLMARK ADIPOGENESIS”, “HALLMARK FATTY ACID METABOLISM”, “KEGG PENTOSE PHOSPHATE”, “HALLMARK MTORC1 SIGNALING”, “HALLMARK/KEGG UNFOLDED PROTEIN RESPONSE”, and “KEGG/HALLMARK OXIDATIVE PHOSPHORYLATION”. In addition, cluster B also showed significant enrichment of the DNA damage response (DDR) pathway and its related pathways, such as “KEGG MISMATCH REPAIR”, “HALLMARK DNA REPAIR”, “KEGG NUCLEOTIDE EXCISION REPAIR”, and “HALLMARK G2M_CHECKPOINT”. The enrichment of cancer-related pathways, such as “HALLMARK TGF BETA SIGNALING” and “HALLMARK MYC TARGETS” (Figure 3C), in cluster B was similarly observed in the cohort of B versus C. In contrast, cluster C showed enrichment of the “KEGG ECM RECEPTOR INTERACTION” pathway and downregulation of the “KEGG B CELL RECEPTOR SIGNALING” pathway (Figure 3D). This suggests that not only the biological processes but also the immune microenvironment may differ among the clusters.

### Differences in Clinical Features and TME Among Three PRG-Related Clusters

**FIGURE 4:**
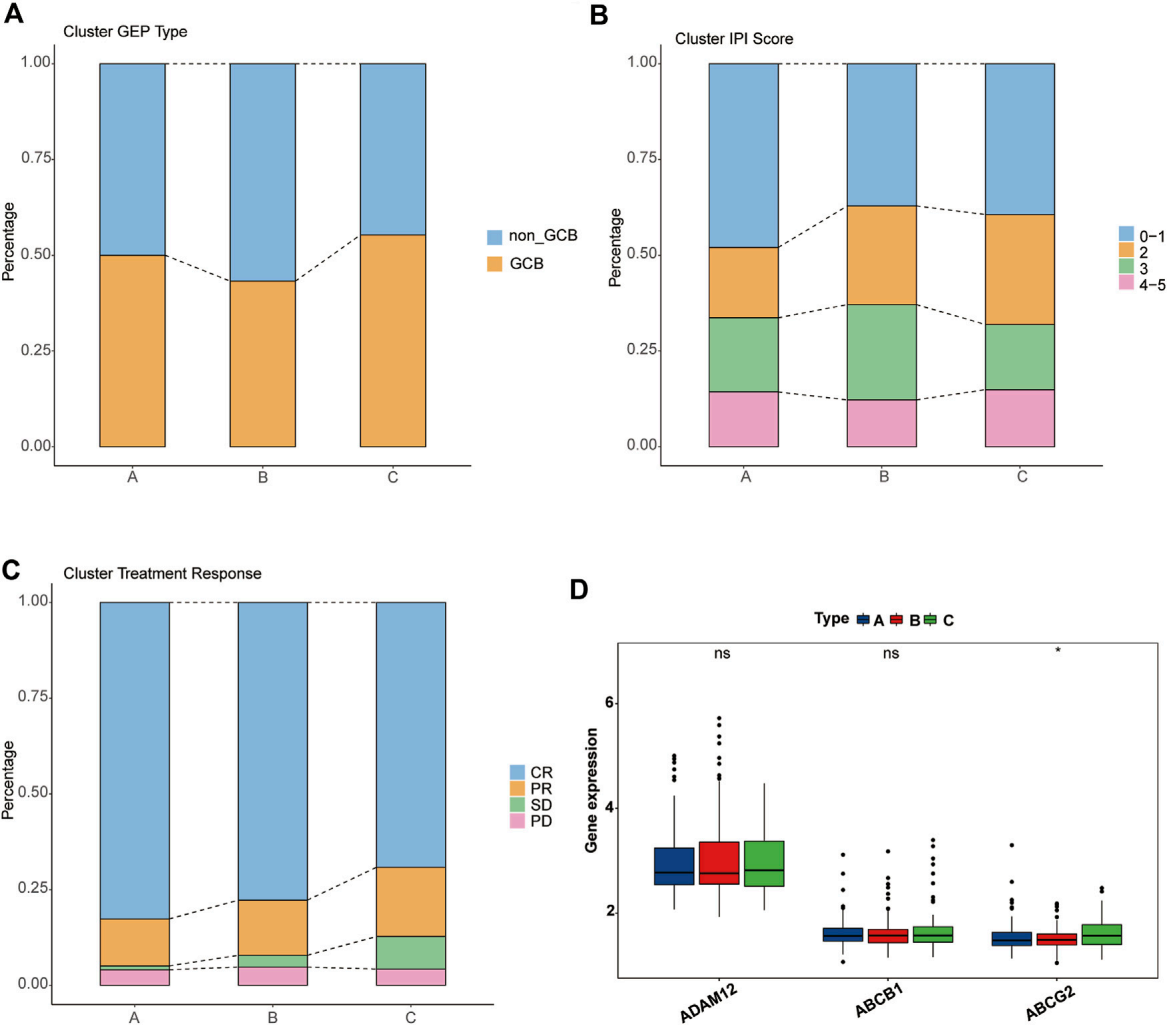
Clinical characteristics of pyroptosis-related clusters. **(A–C)** Differences in the proportion of GEP phenotypes, IPI score, and RCHOP regimen responses among the three clusters. Different colors represent different subtypes, and the column height corresponds to the proportion. **(A)** GEP type. **(B)** IPI score. **(C)** RCHOP regimen response. **(D)** Comparison of the drug-resistance gene expression among clusters A, B, and C.

Next, we analyzed the clinical features of the clusters. Unexpectedly, cluster C, which had the worst survival, showed a higher proportion of the GCB type, whereas cluster B, which was intermediate between A and C, possessed the highest proportion of the non-GCB type (Figure 4A). Regarding the IPI score, cluster A, which had the best survival, showed the highest proportion in the low-risk group (IPI score: 0–1). Compared with cluster B, cluster C accounts for more in low IPI scores (Figure 4B). Given that the dataset GSE31312 as the RCHOP treatment cohort, we then explored the response of three clusters to the treatment regimen. These findings seemed to explain the difference in the survival among the three clusters, such cluster A showing the highest complete remission (CR) rate, cluster B the next highest, and cluster C the worst response to RCHOP treatment (Figure 4C).

We then verified several previously reported genes associated with resistance to the RCHOP regimen and found no significant difference in the expression of “ADAM12” or “ABCB1” among the three clusters, but “ABCG2” was significantly overexpressed in cluster C (Figure 4D) (Ohsawa et al., 2005; Kim et al., 2009; Yin et al., 2017).

**FIGURE 5:**
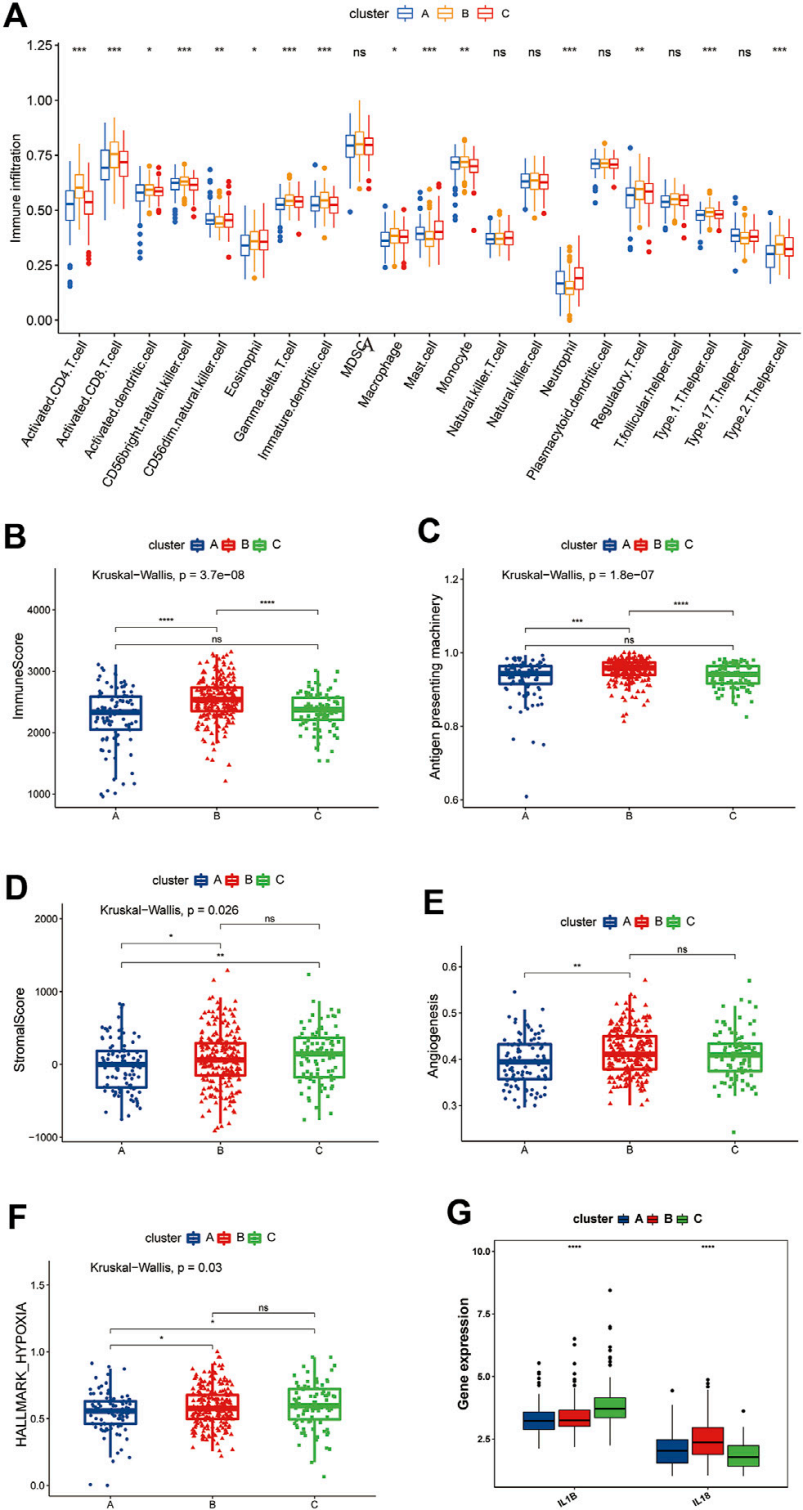
Differences in the TME among the three pyroptosis-related clusters. **(A)** Differences among the three clusters in the abundance of infiltrating immune cells. The blue box, the cluster A; The yellow box, the cluster B; The red box, the cluster C. **(B)** Differences in ImmuneScore among the pyroptosis-related clusters. **(C)** Differences in the APM signature score among the pyroptosis-related clusters. **(D)** Differences in the stromal score among the pyroptosis-related clusters. **(E)** Differences in the angiogenesis signature score among the pyroptosis-related clusters. **(F)** Differences in the hypoxia signature score among the pyroptosis-related clusters. **(G)** Comparison of the IL-1β and IL-18 expression among the pyroptosis-related clusters. **(B–G)** The blue box represents cluster A, the red box represents cluster B, and the green box represents cluster C.

GSVA enrichment analysis suggested that there were differences in the immune microenvironment status among the three clusters. Through an ssGSEA analysis, we found that cluster B had strong adaptive immune activation, including CD8^+^ and CD4^+^ T cells, and it had the highest Treg cell infiltration among the three clusters. Regarding innate immune cells, such as NK T cells, NK cells, and MDSC cells, there were no significant differences among the three clusters (Figure 5A).

We then used the “ESTIMATE” package to score the immunity and stromal of the three clusters. As shown in Figure 5B, cluster B had the highest immune score, which is consistent with the results of the ssGSEA, proving that the TME of cluster B had greater immune cell infiltration than that of the other clusters. Incidentally, we also evaluated the differences antigen-presenting machinery (APM) among the three clusters, which has been shown to be significantly associated with the T cell infiltration score (TIS) (Şenbabaoğlu et al., 2016); the results remained consistent with the former, with cluster B being significantly higher than clusters A and C, while no significant differences were observed between the remaining two clusters (Figure 5C). However, patients in cluster B did not derive a consequential survival benefit. Tumors with the immune-excluded phenotype show infiltration of a large number of immune cells, but due to stromal entrapment, these immune cells are only distributed close to tumor cells and are unable to penetrate the stroma to exert their effect. We therefore also determined the matrix score of the three clusters. A significant difference was noted, which was consistent with the prognosis trend. Cluster A’s stromal scores were smaller than those of clusters B and C, whereas no significant differences were observed between clusters B and C (Figure 5D).

One of the characteristics of the immune-excluded phenotype TME is active angiogenesis (Hegde et al., 2016), so we evaluated the angiogenesis pathways using the ssGSEA algorithm and compared the findings among the three clusters. We found that cluster B had a significantly higher angiogenesis score than cluster A but no significant difference from cluster C was noted (Figure 5E). We also found that clusters B and C had higher levels of hypoxia than cluster A (Figure 5F). IL-1β and IL-18 are the two most important inflammatory mediators of pyroptosis, and we found that IL-1β was highly expressed in cluster C, while IL-18 was highly expressed in cluster B (Figure 5G). Therefore, we speculate that the immune cell activities in cluster B were suppressed by inhibitory cellular, molecular, and other components of the TME and thus were unable to exert their pro-survival effects.

Based on the above findings, we found that the three pyroptosis-related clusters have significantly different TME situations. Cluster B, with pyroptosis-related inflammation dominated by IL-18, closely resembles the immune-excluded phenotype, while cluster C is more consistent with an immune-desert phenotype, with pyroptosis-related inflammation dominated by IL-1β. Cluster A seems characterized by a low pyroptosis-related inflammatory phenotype.

### Identification of Survival-Related PRGs

**FIGURE 6:**
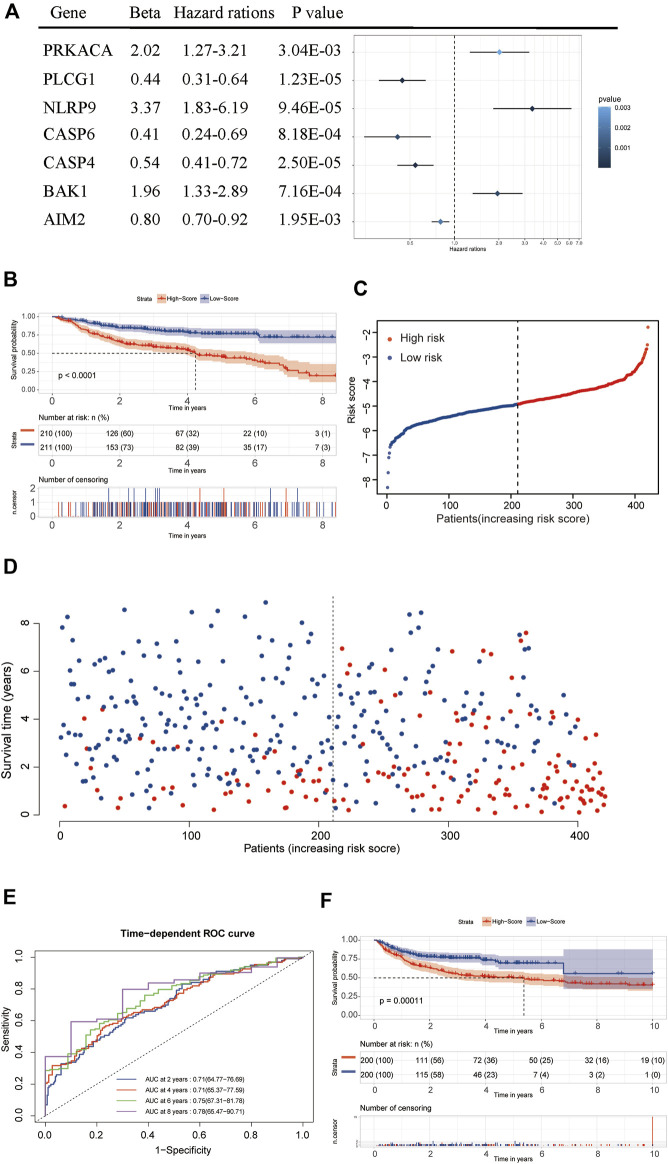
Establishment and validation of the seven-gene prognostic signature. **(A)** Forrest plot of the multivariate Cox regression analysis of seven genes. **(B)** A Kaplan–Meier survival analysis in the training dataset. **(C,D)** The distribution of risk scores and living status of DLBCL patients in the training dataset. **(E)** An ROC curve analysis of the signature. **(F)** A Kaplan–Meier survival analysis in the validation dataset.

After filtering and sorting the data, a total of 421 patients in the GSE31312 dataset met the criteria for inclusion in the survival analysis. A univariate Cox regression analysis showed that a total of 18 genes were significantly associated with the prognosis of DLBCL patients (*p* < 0.05). The results of the univariate analysis of these genes are shown in Table 1. A prognostic signature comprising seven genes—PRKACA, PLCG1, NLRP9, CASP6, CASP4, BAK1, and AIM2—was developed by a Lasso-penalized Cox analysis and stepwise Cox analysis. Based on the hazard ratio (HR), the downregulated PLCG1, CASP6, CASP4, and AIM2 were considered tumor suppressors, whereas the upregulated PRKACA, NLRP9, and BAK1 were regarded as oncogenes (Figure 6A).

**TABLE 1:** Results of a univariate Cox analysis for differential PRGs.

Gene	Hazard ratio	95% CI	*p*-value
TNF	0.53	0.36–0.8	0.002
PYCARD	0.62	0.43–0.91	0.013
PRKACA	1.96	1.28–2.99	0.002
PLCG1	0.57	0.39–0.84	0.004
NOD2	0.61	0.4–0.93	0.023
NLRP9	3.36	1.88–6	0
NLRP6	1.59	1.01–2.5	0.047
NLRP1	1.76	1.21–2.56	0.003
NLRC4	1.58	1.11–2.24	0.01
NAIP	0.78	0.62–0.97	0.029
IL18	0.74	0.58–0.94	0.013
GPX4	0.62	0.44–0.86	0.005
CASP8	1.38	1.05–1.81	0.02
CASP6	0.53	0.32–0.87	0.012
CASP4	0.54	0.42–0.71	0
BAX	0.76	0.6–0.95	0.018
BAK1	2.03	1.34–3.09	0.001
AIM2	0.88	0.78–0.99	0.035

CI, confidence interval.

### Establishment of the Seven-Gene Prognostic Signature and Validation of the Prognostic Performance.

We used the following formula to calculate the risk score:

[(Exp PRKACA × (0.70221) + (Exp PLCG1 × (−0.81499) + Exp NLRP9 × (1.21405) + Exp CASP6× (−0.88874) + Exp CASP4 × (−0.61049) + Exp BAK1 × (0.67189) + Exp AIM2 × (−0.21797)]

The median risk score was then used as the critical value for risk grouping. Patients from the training dataset were stratified into two groups. The Kaplan-Meier survival curves showed a significantly better OS in the low-risk group than in the high-risk group (*p* < 0.0001) (Figures 6B–D). The time-dependent ROC curve was used to determine the prognostic value of the signature. The AUCs for 2-, 4-, 6-, and 8-years OS predictions were 0.71, 0.71, 0.75, and 0.78, respectively, which were all higher than 0.7, proving that the risk score signature had favorable prognostic ability in the training dataset (Figure 6E).

Internal validation of the signature using the bootstrapping method showed that it was reasonably stable (Supplementary Figures S2A–D). The predictive performance of the seven-gene signature was externally validated in the GSE10846 dataset. Kaplan Meier curves showed that the OS remained significantly different between risk groups in the validation dataset (Figure 6F). The prognosis of the high-risk group was significantly worse than that of the low-risk group (*p* < 0.05). External validation showed that the seven-gene signature performed well in predicting the OS in patients with DLBCL.

### Building and Validating a Predictive Nomogram

**FIGURE 7:**
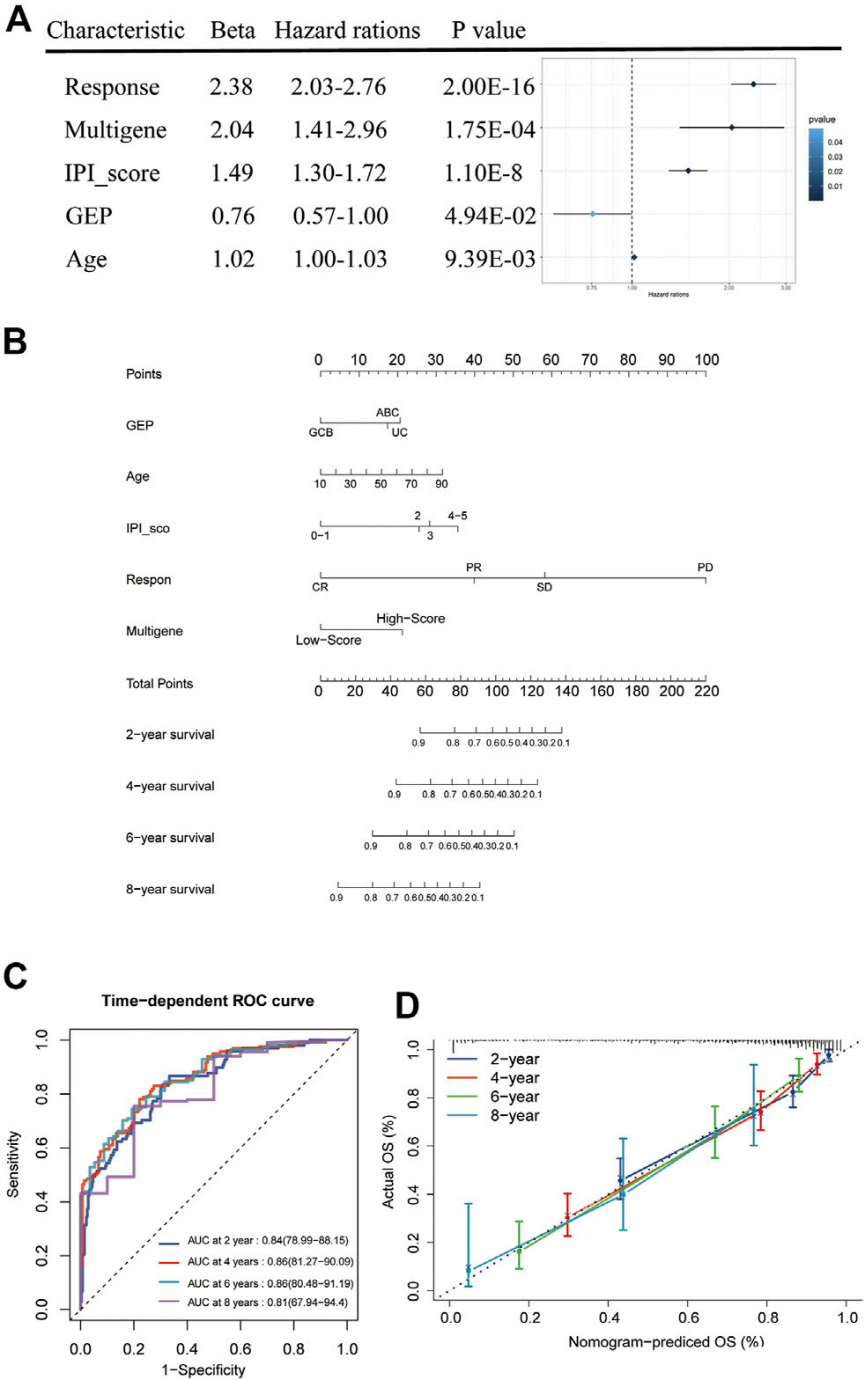
Nomogram construction and prognostic value of the signature. **(A)** Forest plot of clinical factors and risk score. **(B)** The nomogram for predicting the survival rate after 2, 4, 6, and 8 years in DLBCL patients. **(C)** The time-dependent ROC analysis of nomogram predicting the survival rate after 2, 4, 6, and 8 years in DLBCL patients. **(D)** Calibration plots of the nomogram.

We combined the risk status with the age, gender, GEP, treatment regimen (RCHOP) and response, and IPI score as candidate predictors and created a prognostic nomogram predicting the OS of DLBCL patients based on the LASSO regression analysis and stepwise Cox regression analysis. The risk status, age, GEP, response, and IPI score were considered to be the final parameters in the nomogram (Figures 7A,B). The AUCs of the OS for 2, 4, 6, and 8 years were 0.84, 0.86, 0.86, and 0.81, respectively, (Figure 7C). The C-index of the nomogram was 0.833 (95% confidence interval [CI]: 0.816–0.85), and the calibration curves revealed that the predicted the OS accorded with the observed OS (Figure 7D). Taken together, these results show that the nomogram has an excellent ability to predict the survival time in DLBCL patients.

### Association of the Pyroptosis Risk Score With Clinical Features and TME

**FIGURE 8:**
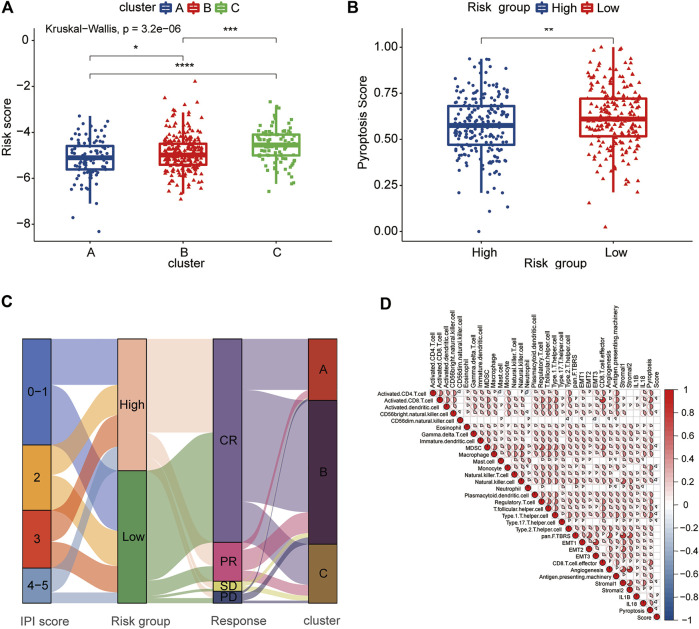
Clinical and TME characteristics of pyroptosis risk score and pyroptosis risk subgroup. **(A)** Comparison of the pyroptosis risk score among the three pyroptosis-related clusters. The blue box represents cluster A, the red box cluster B and the green box cluster C. **(B)** Comparison of the pyroptosis signature score between the two pyroptosis risk groups. Blue box, high-risk group; red box, low-risk group. **(C)** Alluvial diagram showing the changes in the IPI score, pyroptosis risk group, RCHOP regimen treatment response and pyroptosis-related clusters. **(D)** Correlation among the TME, expression of IL-1β and IL-18, pyroptosis signature score and pyroptosis score. Red represents a positive correlation, and blue represents a negative correlation. The color depth represents the strength of the correlation.

The pyroptosis risk score gradually increased among the three clusters of A, B, and C, which was consistent with the difference in the prognosis between the clusters (Figure 8A). The low-risk group had a higher pyroptosis signature score than the high-risk group (Figure 8B). We further analyzed the relationship between the pyroptosis risk group and IPI score, response to treatment and PRG-related clusters and visualized them using an alluvial diagram (Figure 8C). It was evident that the low-risk group had lower IPI scores and better treatment responses than the high-risk group. The above analyses suggested that the low-risk group was significantly associated with more favorable clinical features for the survival than the high-risk group.

To evaluate the correlation between the TME and pyroptosis in DLBCL, we included the pyroptosis risk score, pyroptosis signature score and TME components, including immune cells, APM, CD8^+^ T cell effort, and stroma-related pathways, in the correlation matrix for the comprehensive analysis. Infiltrating immune cells, such as activated CD8^+^ T cells and NK cells, showed a negative correlation with the pyroptosis risk score, while stromal pathways, such as pan-F-TBRS, EMT1-3 and stromal 2, were not correlated (Figure 8D). Notably, the prognostically favorable stromal 1 signature was also negatively correlated with the pyroptosis risk score. The pyroptosis signature score showed an extremely strong correlation with the TME, associated with either adaptive or innate immunity, and stromal components.

## Discussion

Pyroptosis is closely related to human diseases, and an increasing number of studies have proven that pyroptosis acts as a “double-edged sword” in human tumors (Xia et al., 2019). As a type of cell death, pyroptosis can inhibit tumor development by inducing tumor death. Knockdown of LncRNA-XIST inhibited non-small-cell lung cancer (NSCLC) progression by triggering pyroptosis via the miR-335/SOD2/ROS signaling pathway (Liu et al., 2019). Overexpressed LncRNA ADAMTS9-AS2 inhibits gastric cancer (GC) progression by regulating miR-223-3p/NLRP3 axis-mediated pyroptosis (Ren et al., 2020). However, these tumor suppressive effects may be attenuated by epigenetic modifications, such as tumor-suppression genes ZDHHC1 and DRD2 (Le et al., 2020; Tan et al., 2021). Multiple signaling pathways and inflammatory mediators are released during pyroptosis and are closely associated with tumorigenesis as well as resistance to chemotherapeutic drugs (Grivennikov et al., 2010). GSDMD, as the major executor of pyroptosis, was concomitantly significantly elevated compared to normal B cells, suggesting that pyroptotic activity is greater in DLBCL. We also observed a significantly higher expression of IL-1β and IL-18 in DLBCL, suggesting that DLBCL is in a strong inflammatory response which may be mediated by pyroptosis. To our surprise, we found that the expression of none of the genes in the GSDM family was associated with the DLBCL prognosis, which may reflect the complex role of inflammation in tumorigenesis (Zhang et al., 2021).

Most previous studies focused on a single or few pyroptosis-related molecules, and recently, attention has been paid to this issue. For example, Ye et al. explored the roles of multiple PRGs in ovarian cancer, such that analysis modalities allow probing the role of this phenotype in disease from a holistic perspective (Ye et al., 2021). In the present study, we identified 3 distinct pyroptosis-associated clusters based on 52 PRGs. We detected significant prognostic differences in patients belonging to different clusters, with patients classified as cluster A having the best prognosis, whereas those in cluster C had the worst prognosis. This suggested heterogeneity in biological pathways among the three different clusters. An analysis of the clinical characteristics of the three clusters showed that cluster A was biased toward more favorable clinical characteristics, such as the GCB phenotype and a low-risk IPI score, than the other clusters. Unexpectedly, cluster C showed a higher proportion of GEP-type patients as well as low-risk IPI score than cluster B, suggesting that these two clinical features cannot fully explain the heterogeneity of pyroptosis-associated clusters.

RCHOP is currently the standard care for pre-treatment DLBCL, and responsiveness to drugs is directly related to the patient prognosis (Tilly et al., 2015). Our analysis of the drug response was consistent with the different prognostic features among the three clusters, as cluster A had the best prognosis and the highest CR rate, while cluster C had a poor prognosis and the lowest CR rate; furthermore, the mRNA level of ABCG2, which is related to doxorubicin resistance, was particularly high in cluster C (Kim et al., 2009). GSVA enrichment analysis showed that cluster B contained a significant activation of DDR processes as well as enrichment of numerous cancer promotion and metabolism-related pathways. DNA repair pathways are critical for the tumor cell survival during exposure to chemotherapy. Overexpression of NER pathway genes by high-risk DLBCL is associated with resistance to the CHOP regimen, with higher DNA repair scores implying a poor prognosis (Bret et al., 2013; Bret et al., 2015). We similarly observed significant oxidative phosphorylation, high activity of the TCA cycle and fatty acid metabolism enrichment in cluster B, findings similar to those observed for OXPHOS-DLBCL by Caro et al. and features that favor the growth and survival of DLBCL and engender resistance to conventional agents that target the BCR signaling axis (Caro et al., 2012). The above results indicated that cluster B was enriched with a large number of poor prognostic pathways, which contributed to the decreased OS of patients in cluster B.

The role of pyroptosis in the TME has been gradually appreciated, and the inflammatory microenvironment it brings about was found to be associated with the proliferation, survival, immunosuppression, and angiogenesis of a variety of tumors (Karki and Kanneganti, 2019). The pre-existing host immune infiltration has a suggestive significance concerning the prognosis of DLBCL patients treated with the RCHOP regimen, which suggests that the difference in the prognosis among our three clusters may have been related to differences in the immune microenvironment (Ansell et al., 2001). Through an immune infiltration analysis, we conformed that the three clusters had obvious heterogeneity in the TME. The characteristics of the three clusters are summarized as follows: Cluster A showed a relatively low abundance of immune cell infiltration but low levels of stromal element infiltration and inflammation compared with clusters B and C; Cluster B had significant adaptive immune activation, a high degree of stromal activation and inflammation dominated by IL-18; Cluster C had a lower level of adaptive immune activation than cluster B but had the highest degree of stromal infiltration among the three clusters as well as inflammation dominated by IL-1β. Baldini et al. found that the P2X7 receptor-NLRP3 inflammasome complex is a promising factor for predicting the development of NHL in Sjogren’s syndrome (SS). The IL-18 levels were higher in SS glands with MALT-NHL, underscoring the importance of IL-18 in the pathogenesis of malignant lymphoproliferative diseases (Baldini et al., 2017). The IL-18 mRNA level was significantly elevated in primary DLBCL, and increasing serum IL-18 levels were also found to be associated with a poor prognosis in DLBCL patients who were treated with the RCHOP regimen (Goto et al., 2011). Further studies revealed that IL-18 can promote proliferation and inhibit apoptosis in lymphoma cells by altering the balance of c-myc/TP53 and Bcl-2/Bax ( Zhao et al., 2017). The study by Lu et al. similarly supported the notion that IL-1β and IL-18 levels are elevated in lymphoma and that blocking them can retard disease progression (Lu et al., 2021). The infiltration of immune cells with high abundance in cluster B was accompanied by significantly elevated stromal infiltration, suggesting that cluster B closely resembles the immune-excluded phenotype (Chen and Mellman, 2017). Despite the presence of a pre-existing immune response, it is unable to exert anti-tumor effects because immune cells are retained in the stromal. We observed a remarkable positive correlation of the pan-F-TBRS signature with both IL-1β and IL18 expression, demonstrating that pyroptosis-induced inflammation is associated with increased infiltration of fibroblasts (Mariathasan et al., 2018). A dense stroma impedes the movement of T cells into the tumor, leading to the appearance of a rejection phenotype in cluster B (Salmon et al., 2012). Cluster B was also notable for the presence of angiogenesis and the infiltration of tumor-associated macrophages (TAMs), both of which play a role in limiting T cell clustering in the vicinity of the tumor (Joyce and Fearon, 2015). Furthermore, the massive infiltration of regulatory T cells, as well as the enrichment of the TGF-β signaling pathway, suggested that the function of the infiltrated T cells in cluster B might be suppressed (Zou, 2006; Massagué, 2008). Given that cluster C had prominent stromal infiltration but no prominent immune cell infiltration, we considered it more consistent with an immune-desert phenotype (Chen and Mellman, 2017). We observed significantly elevated NLRP3 levels in cluster C, which suppressed the anti-proliferative effects of dexamethasone. Inflammasome NLRP3 activation promotes lymphoma cell proliferation and inhibits apoptosis by upregulating c-myc and BCL2 and downregulating TP53, and Bax, which in turn reduces the antitumor effect of dexamethasone (Zhao et al., 2017). IL-1β, as an additional pyroptosis important inflammatory factor, was found to be significantly elevated in cluster C. The IL-1β pathways promote tumor growth and metastasis in breast cancer models, and tumor progression is associated with increased levels of IL-1β at primary and metastatic sites, an effect associated with activation of the inflammasome NLRP3 (Guo et al., 2016). Blocking IL-1β reverses the immunosuppression in mouse breast cancer and promotes tumor cell regression (Kaplanov et al., 2019). High levels of immune cell infiltration were not seen for cluster A, and we were unable to classify this as an immunoinflammatory phenotype; however, we also did not detect pyroptotic inflammation. This may have been due to the fact that neither IL-18 nor IL-1β was appreciably elevated in cluster A, a finding associated with an optimal response to therapy, ultimately leading to a better prognostic outcome.

Heterogeneity in pyroptosis-associated clusters is an among-group difference, but inter-individual differences are also important to consider. Therefore, we designed a pyroptosis risk scoring system for individual patient evaluation. We found that the scores were higher in cluster C than in cluster A, where inflammation was not intense. This indicates that the scoring system can assess not only the inter-individual heterogeneity of pyroptosis but also the pattern of pyroptosis among individuals, confirming its reliability. In parallel, we also conducted an analysis of the correlation of this scoring system with clinical features and demonstrated showed high scores were associated with worse clinical characteristics.

In summary, our analysis suggests that pyroptosis may have different patterns of action in DLBCL, and high pyroptosis levels did not confer a more favorable survival prognosis, mainly due to the inflammation induced by pyroptosis. This observation fits the current definition of the role of pyroptosis in tumors as a “double-edged sword”. Inflammation can promote tumorigenesis and antitumor immunity at all stages of tumor development. The ultimate effect of inflammation may be related to whether tumor promotion or inhibition dominates and also depend on differences in the host inflammatory status and immunity (Zhang et al., 2021). Considering the inter-individual heterogeneity of pyroptosis, we developed a pyroptosis-gene risk score signature that was able to effectively identify patients at high and low risk. Based on this risk signature and the clinical characteristics of DLBCL, a nomogram was constructed and used to excellently predict the prognosis of DLBCL patients. Our findings have important suggestive implications for clinical management. For patients with a low level of pyroptosis-related inflammation, the RCHOP regimen may be beneficial, whereas a low response rate and resistance to RCHOP treatment may be points of concern in patients with a high IL-1β expression. Patients with elevated IL-18 levels have a characteristic immune-excluded phenotype, suggesting that overcoming T cell suppression and rejection may enable such patients to benefit from immunotherapy (Joyce and Fearon, 2015).

We must acknowledge that this study is not prospective, which is an inescapable limitation. Further experiments are needed to verify the mechanism underlying the actions of pyroptosis-related molecules. This is a great challenge for us, but the ability of current single-cell sequencing technologies to clarify the TME is encouraging. The establishment of prospective cohorts and reliance on single-cell sequencing techniques to mine pyroptosis-associated immune microenvironment features are key directions that will be pursued in our follow-up studies.

## Conclusion

Our study revealed the complicated role of pyroptosis in DLBCL, and differences in pyroptosis patterns may responsible for the tumor and tumor microenvironment heterogeneity. The contribution of pyroptosis to immune microenvironment shaping is nonnegligible, and properly recognizing and inducing pyroptosis to generate a tumor suppressor microenvironment is a worthy direction to explore. Furthermore, a comprehensive assessment of individual pyroptosis patterns and signatures based on pyroptosis may assist in establishing personalized treatment of DLBCL patients.

## Data Availability

The original contributions presented in the study are included in the article/Supplementary Material, further inquiries can be directed to the corresponding authors.

## Supplementary Material

Supplementary Figure 1
**(A)** The forest plot of univariate Cox regression of PRGs. The darker the dot color, the more significant the solution result is. **(B,C)** Identification of pyroptosis-associated clusters. The 421 DLBCL patients from the GSE31312 dataset were divided into 3 clusters based on the consensus clustering matrix (k = 3).

Supplementary Figure 2
**(A–D)** Results of internal verification of the seven-gene signature, using the bootstrapping method. **(E,F)** A Lasso analysis of the prognostic PRGs in DLBCL.

## Abbreviations

DLBCL, Diffuse large B-cell lymphoma; B-NHL, B-cell non-Hodgkin lymphoma; TME, Tumor immune microenvironment; PRGs, Pyroptosis-related genes; RCHOP, rituximab plus cyclophosphamide, doxorubicin, vincristine, prednisone; CNV, copy number variation; NLRP3, NLR Family Pyrin Domain Containing 3; IL-1β, Interleukin 1 beta; IL-18, Interleukin 18; GSDMD, gasdermin D; EMT, epithelial-mesenchymal transition; GEO, Gene Expression Omnibus; TCGA, The Cancer Genome Atlas; TCGA-DLBC, The Cancer Genome Atlas Project: Lymphoid Neoplasm Diffuse Large B-cell Lymphoma; GEP, gene expression profiling; IPI, International Prognostic Index; ssGSEA: single-sample gene-set enrichment analysis; APM, antigen presenting machinery; OS, overall survival; DDR, DNA damage response; pan-F-TBRS, pan-fibroblast TGFβ response signature; CR, complete remission.
