# Supplementary figures and images for: The Dark Side of Pyroptosis of Diffuse Large B-Cell Lymphoma in B-Cell Non-Hodgkin Lymphoma: Mediating the Specific Inflammatory Microenvironment
Source: Front Cell Dev Biol. 2021 Nov 5;9:779123. doi: 10.3389/fcell.2021.779123 (PMC8602351; doi:10.3389/fcell.2021.779123)

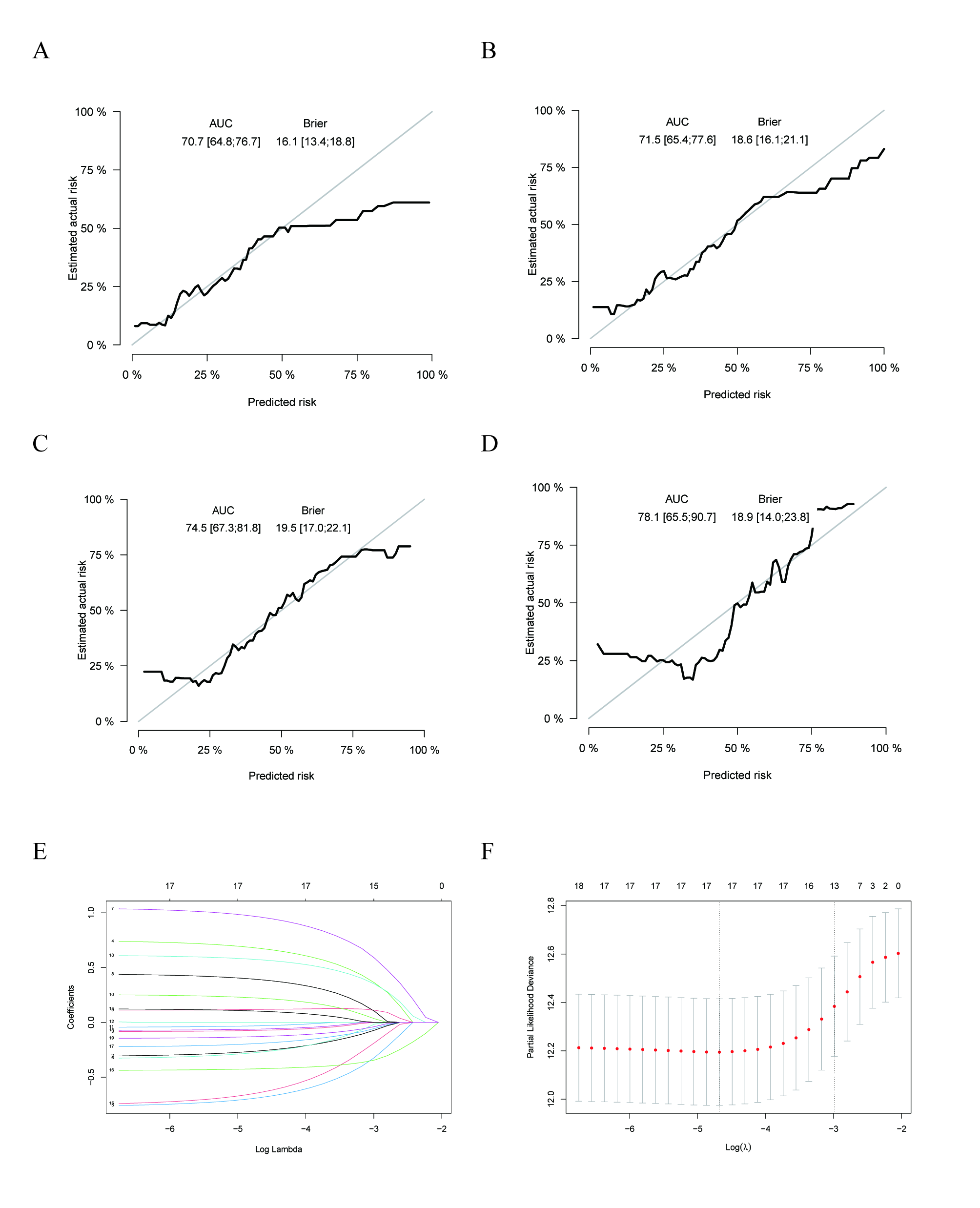

Supplement: Supplementary file 1 [file Image2.TIF]

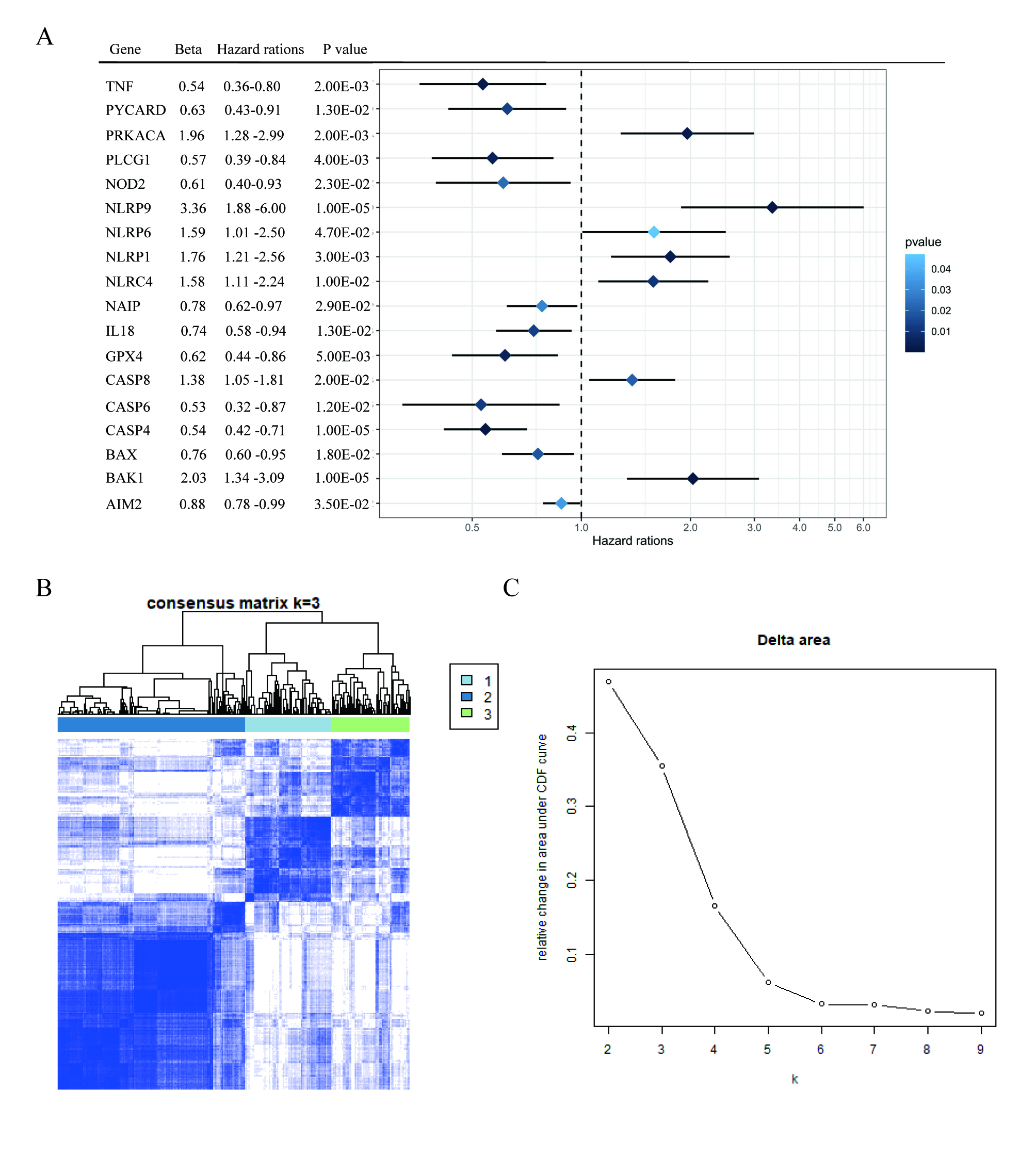

Supplement: Supplementary file 2 [file Image1.TIF]
